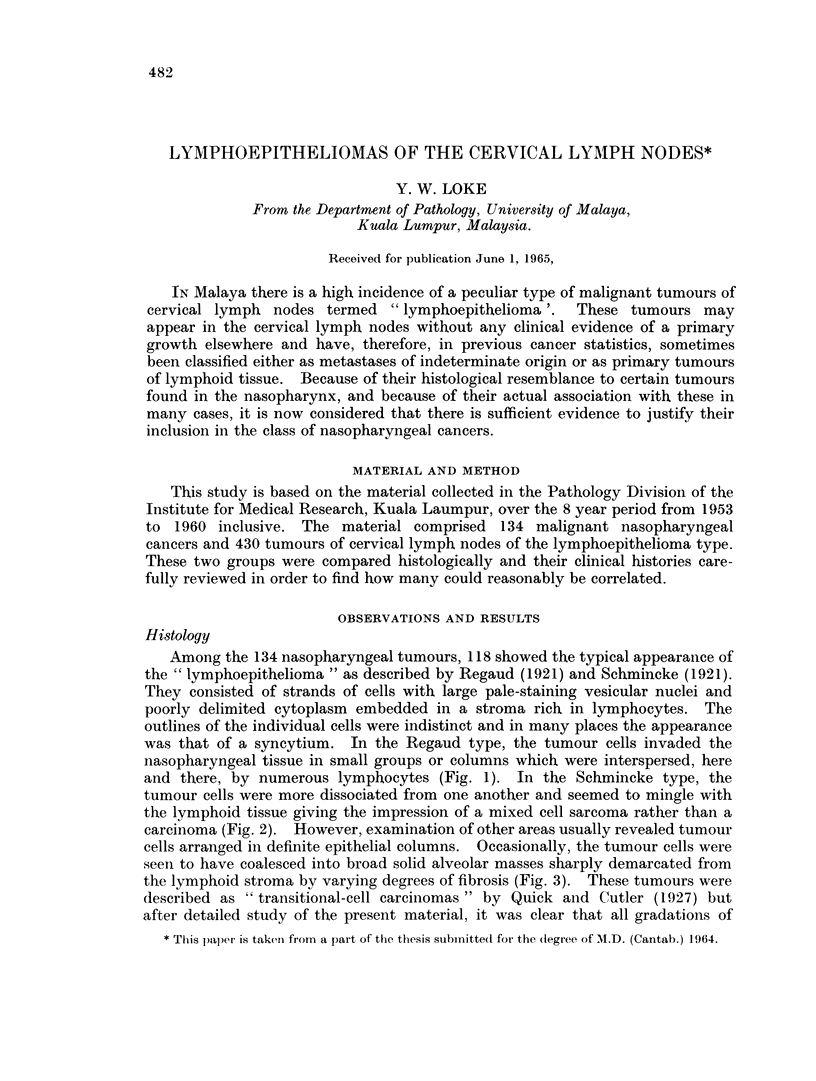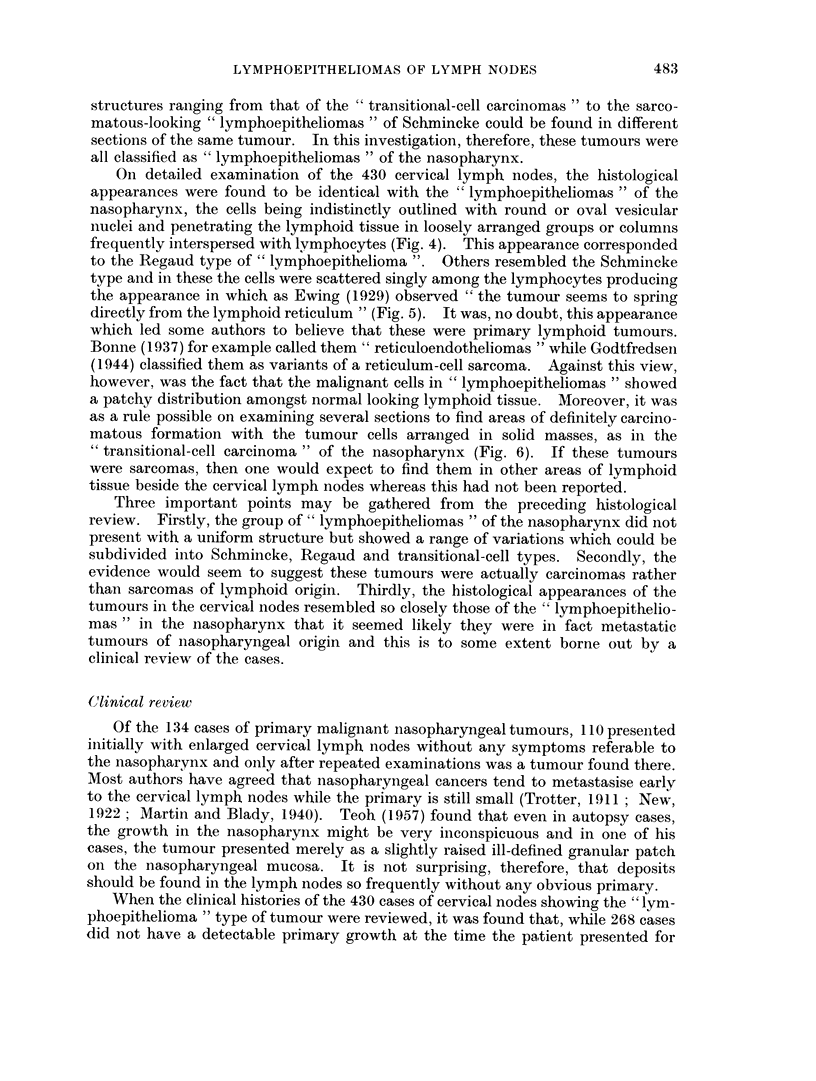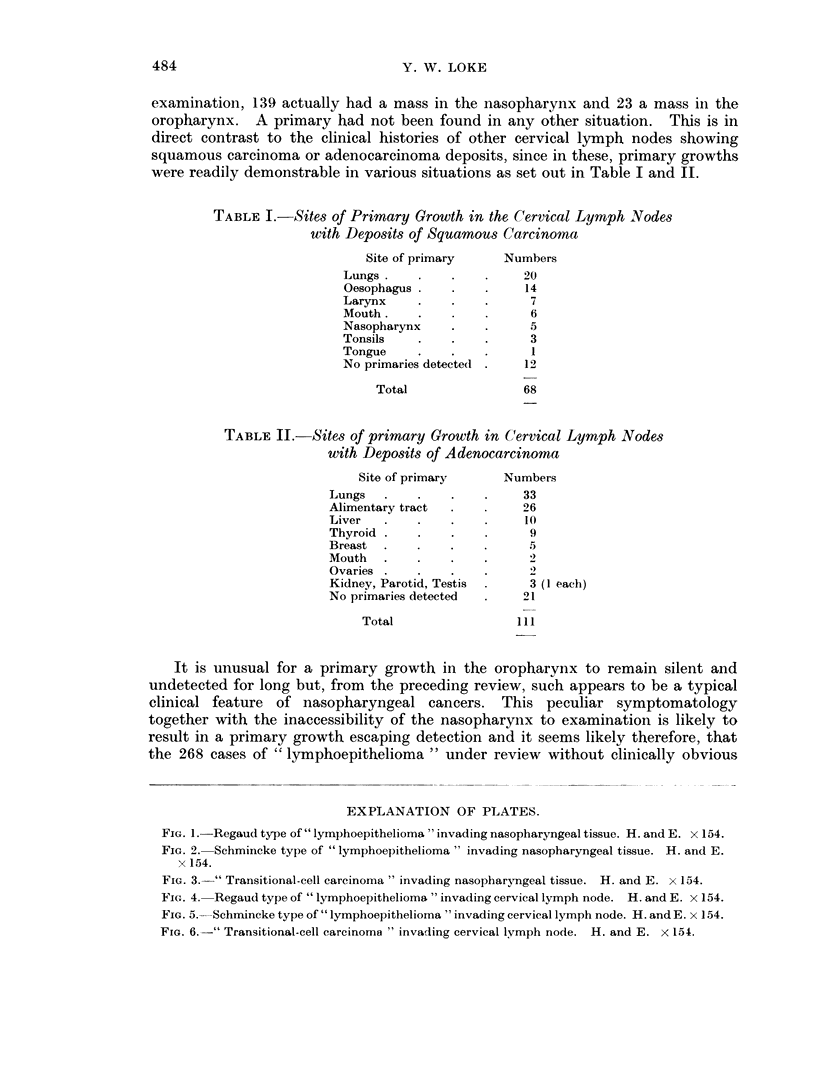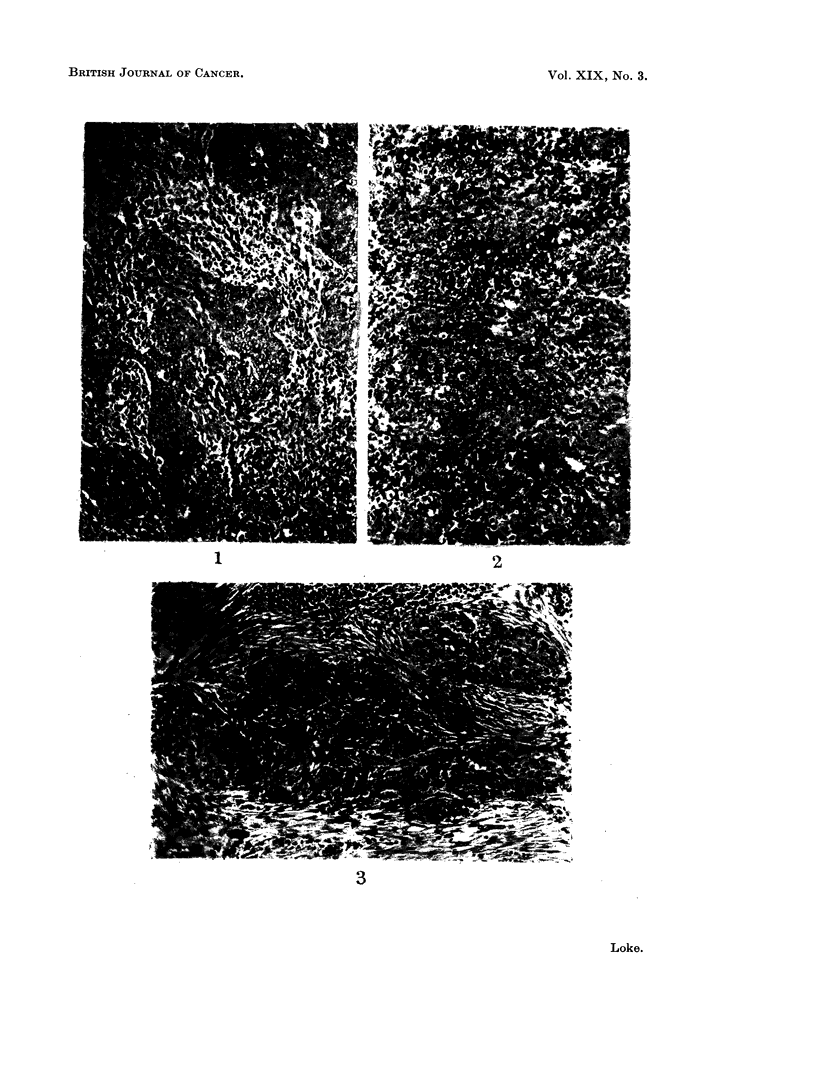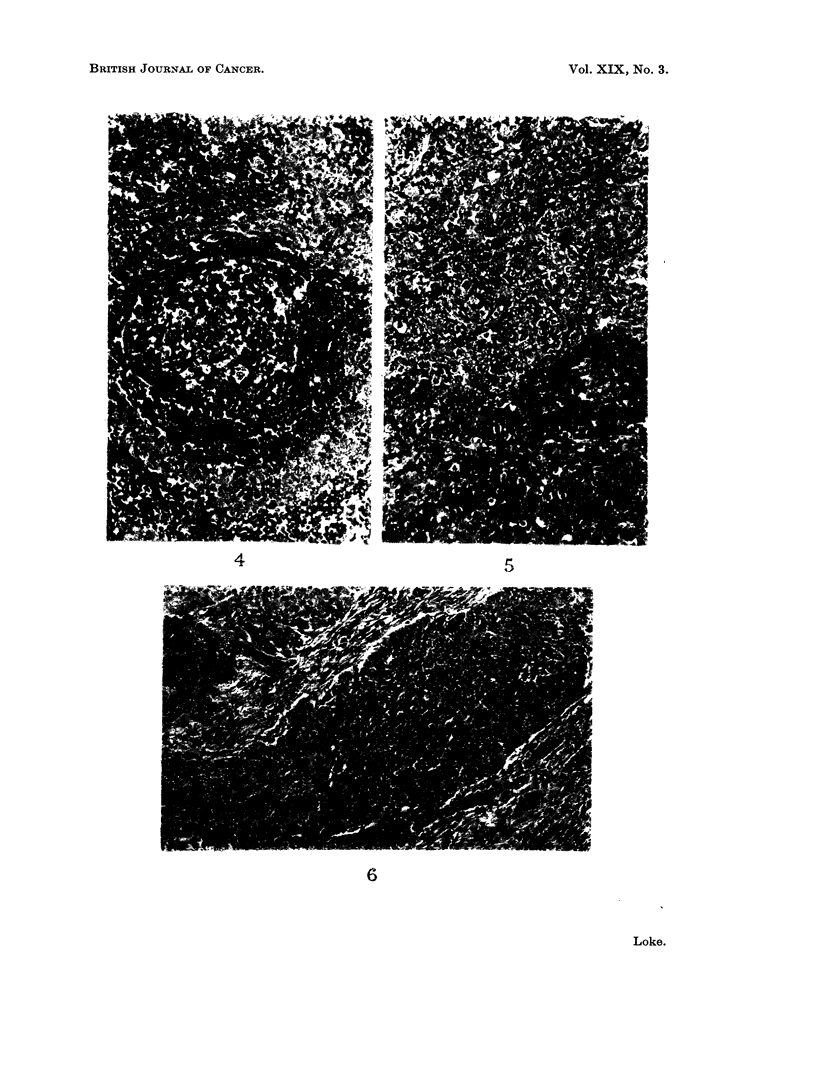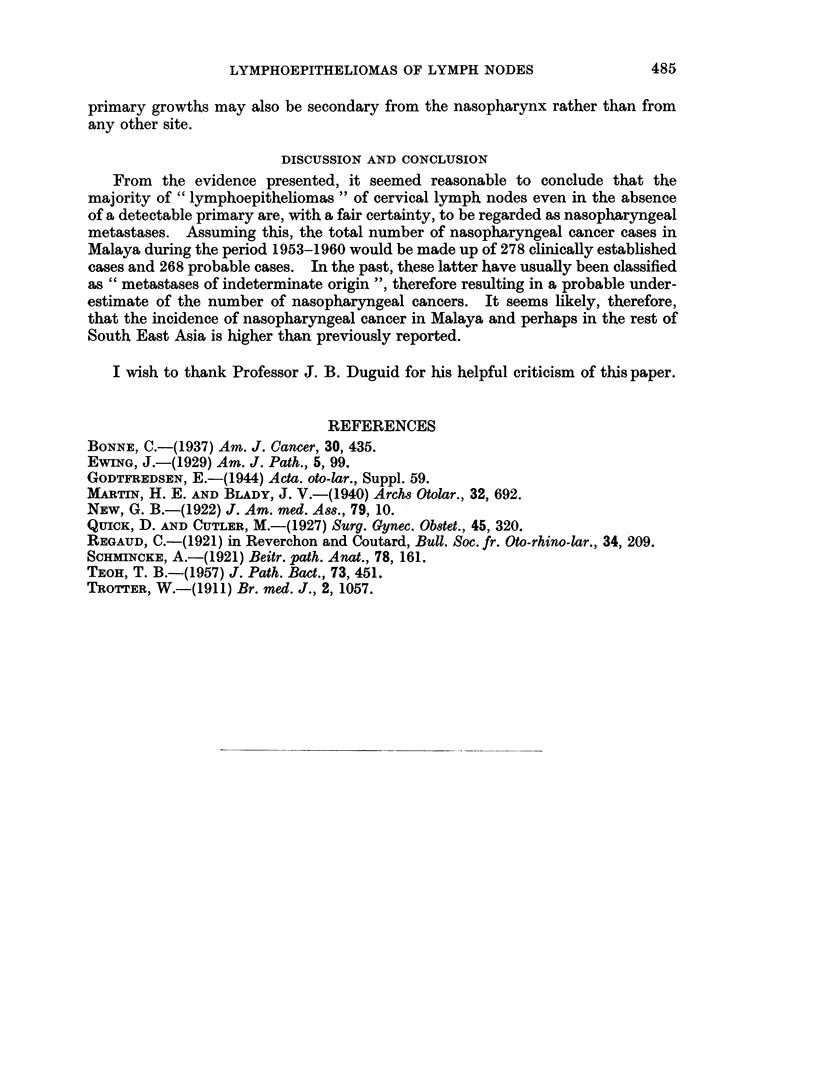# Lymphoepitheliomas of the cervical lymph nodes.

**DOI:** 10.1038/bjc.1965.56

**Published:** 1965-09

**Authors:** Y. W. Loke

## Abstract

**Images:**


### LYMPHOEPITHELIOMAS OF THE CERVICAL LYMPH NODES*

Y. W. LOKE

*From the Department of Pathology, University of Malaya, Kuala Lumpur, Malaysia.*

Received for publication June 1, 1965,

In Malaya there is a high incidence of a peculiar type of malignant tumours of cervical lymph nodes termed " lymphoepithelioma '. These tumours may appear in the cervical lymph nodes without any clinical evidence of a primary growth elsewhere and have, therefore, in previous cancer statistics, sometimes been classified either as metastases of indeterminate origin or as primary tumours of lymphoid tissue. Because of their histological resemblance to certain tumours found in the nasopharynx, and because of their actual association with these in many cases, it is now considered that there is sufficient evidence to justify their inclusion in the class of nasopharyngeal cancers.

### MATERIAL AND METHOD

This study is based on the material collected in the Pathology Division of the Institute for Medical Research, Kuala Laumpur, over the 8 year period from 1953 to 1960 inclusive. The material comprised 134 malignant nasopharyngeal cancers and 430 tumours of cervical lymph nodes of the lymphoepithelioma type. These two groups were compared histologically and their clinical histories carefully reviewed in order to find how many could reasonably be correlated.

### OBSERVATIONS AND RESULTS

### *Histology*

Among the 134 nasopharyngeal tumours, 118 showed the typical appearance of the " lymphoepithelioma " as described by Regaud (1921) and Schmincke (1921). They consisted of strands of cells with large pale-staining vesicular nuclei and poorly delimited cytoplasm embedded in a stroma rich in lymphocytes. The outlines of the individual cells were indistinct and in many places the appearance was that of a syncytium. In the Regaud type, the tumour cells invaded the nasopharyngeal tissue in small groups or columns which were interspersed, here and there, by numerous lymphocytes (Fig. 1). In the Schmincke type, the tumour cells were more dissociated from one another and seemed to mingle with the lymphoid tissue giving the impression of a mixed cell sarcoma rather than a carcinoma (Fig. 2). However, examination of other areas usually revealed tumour cells arranged in definite epithelial columns. Occasionally, the tumour cells were seen to have coalesced into broad solid alveolar masses sharply demarcated from the lymphoid stroma by varying degrees of fibrosis (Fig. 3). These tumours were described as " transitional-cell carcinomas " by Quick and Cutler (1927) but after detailed study of the present material, it was clear that all gradations of

* This paper is taken from a part of the thesis submitted for the degree of M.D. (Cantab.) 1964.

structures ranging from that of the " transitional-cell carcinomas " to the sarcomatous-looking " lymphoepitheliomas " of Schmincke could be found in different sections of the same tumour. In this investigation, therefore, these tumours were all classified as " lymphoepitheliomas " of the nasopharynx.

On detailed examination of the 430 cervical lymph nodes, the histological appearances were found to be identical with the " lymphoepitheliomas " of the nasopharynx, the cells being indistinctly outlined with round or oval vesicular nuclei and penetrating the lymphoid tissue in loosely arranged groups or columns frequently interspersed with lymphocytes (Fig. 4). This appearance corresponded to the Regaud type of " lymphoepithelioma ". Others resembled the Schmincke type and in these the cells were scattered singly among the lymphocytes producing the appearance in which as Ewing (1929) observed " the tumour seems to spring directly from the lymphoid reticulum " (Fig. 5). It was, no doubt, this appearance which led some authors to believe that these were primary lymphoid tumours. Bonne (1937) for example called them " reticuloendotheliomas " while Godtfredsen (1944) classified them as variants of a reticulum-cell sarcoma. Against this view, however, was the fact that the malignant cells in " lymphoepitheliomas " showed a patchy distribution amongst normal looking lymphoid tissue. Moreover, it was as a rule possible on examining several sections to find areas of definitely carcinomatous formation with the tumour cells arranged in solid masses, as in the " transitional-cell carcinoma " of the nasopharynx (Fig. 6). If these tumours were sarcomas, then one would expect to find them in other areas of lymphoid tissue beside the cervical lymph nodes whereas this had not been reported.

Three important points may be gathered from the preceding histological review. Firstly, the group of " lymphoepitheliomas " of the nasopharynx did not present with a uniform structure but showed a range of variations which could be subdivided into Schmincke, Regaud and transitional-cell types. Secondly, the evidence would seem to suggest these tumours were actually carcinomas rather than sarcomas of lymphoid origin. Thirdly, the histological appearances of the tumours in the cervical nodes resembled so closely those of the " lymphoepitheliomas " in the nasopharynx that it seemed likely they were in fact metastatic tumours of nasopharyngeal origin and this is to some extent borne out by a clinical review of the cases.

*Clinical review*

Of the 134 cases of primary malignant nasopharyngeal tumours, 110 presented initially with enlarged cervical lymph nodes without any symptoms referable to the nasopharynx and only after repeated examinations was a tumour found there. Most authors have agreed that nasopharyngeal cancers tend to metastasise early to the cervical lymph nodes while the primary is still small (Trotter, 1911 ; New, 1922 ; Martin and Blady, 1940). Teoh (1957) found that even in autopsy cases, the growth in the nasopharynx might be very inconspicuous and in one of his cases, the tumour presented merely as a slightly raised ill-defined granular patch on the nasopharyngeal mucosa. It is not surprising, therefore, that deposits should be found in the lymph nodes so frequently without any obvious primary.

When the clinical histories of the 430 cases of cervical nodes showing the "lymphoepithelioma " type of tumour were reviewed, it was found that, while 268 cases did not have a detectable primary growth at the time the patient presented for examination, 139 actually had a mass in the nasopharynx and 23 a mass in the oropharynx. A primary had not been found in any other situation. This is in direct contrast to the clinical histories of other cervical lymph nodes showing squamous carcinoma or adenocarcinoma deposits, since in these, primary growths were readily demonstrable in various situations as set out in Table I and II.

TABLE I.—*Sites of Primary Growth in the Cervical Lymph Nodes with Deposits of Squamous Carcinoma*

| Site of primary | Numbers |
|---|---|
| Lungs | 20 |
| Oesophagus | 14 |
| Larynx | 7 |
| Mouth | 6 |
| Nasopharynx | 5 |
| Tonsils | 3 |
| Tongue | 1 |
| No primaries detected | 12 |
| Total | 68 |

TABLE II.—*Sites of primary Growth in Cervical Lymph Nodes with Deposits of Adenocarcinoma*

| Site of primary | Numbers |
|---|---|
| Lungs | 33 |
| Alimentary tract | 26 |
| Liver | 10 |
| Thyroid | 9 |
| Breast | 5 |
| Mouth | 2 |
| Ovaries | 2 |
| Kidney, Parotid, Testis | 3 (1 each) |
| No primaries detected | 21 |
| Total | 111 |

It is unusual for a primary growth in the oropharynx to remain silent and undetected for long but, from the preceding review, such appears to be a typical clinical feature of nasopharyngeal cancers. This peculiar symptomatology together with the inaccessibility of the nasopharynx to examination is likely to result in a primary growth escaping detection and it seems likely therefore, that the 268 cases of " lymphoepithelioma " under review without clinically obvious

EXPLANATION OF PLATES.

FIG. 1.—Regaud type of " lymphoepithelioma " invading nasopharyngeal tissue. H. and E. ×154.

FIG. 2.—Schmincke type of " lymphoepithelioma " invading nasopharyngeal tissue. H. and E. ×154.

FIG. 3.—" Transitional-cell carcinoma " invading nasopharyngeal tissue. H. and E. ×154.

FIG. 4.—Regaud type of " lymphoepithelioma " invading cervical lymph node. H. and E. ×154.

FIG. 5.—Schmincke type of " lymphoepithelioma " invading cervical lymph node. H. and E. ×154.

FIG. 6.—" Transitional-cell carcinoma " invading cervical lymph node. H. and E. ×154.

1 2 3

Loke.

4

5

6

Loke.

primary growths may also be secondary from the nasopharynx rather than from any other site.

### DISCUSSION AND CONCLUSION

From the evidence presented, it seemed reasonable to conclude that the majority of " lymphoepitheliomas " of cervical lymph nodes even in the absence of a detectable primary are, with a fair certainty, to be regarded as nasopharyngeal metastases. Assuming this, the total number of nasopharyngeal cancer cases in Malaya during the period 1953–1960 would be made up of 278 clinically established cases and 268 probable cases. In the past, these latter have usually been classified as " metastases of indeterminate origin ", therefore resulting in a probable underestimate of the number of nasopharyngeal cancers. It seems likely, therefore, that the incidence of nasopharyngeal cancer in Malaya and perhaps in the rest of South East Asia is higher than previously reported.

I wish to thank Professor J. B. Duguid for his helpful criticism of this paper.

### REFERENCES

Bonne, C.—(1937) *Am. J. Cancer*, **30**, 435.
Ewing, J.—(1929) *Am. J. Path.*, **5**, 99.
Godtfredsen, E.—(1944) *Acta. oto-lar.*, Suppl. 59.
Martin, H. E. and Blady, J. V.—(1940) *Archs Otolar.*, **32**, 692.
New, G. B.—(1922) *J. Am. med. Ass.*, **79**, 10.
Quick, D. and Cutler, M.—(1927) *Surg. Gynec. Obstet.*, **45**, 320.
Regaud, C.—(1921) in Reverchon and Coutard, *Bull. Soc. fr. Oto-rhino-lar.*, **34**, 209.
Schmincke, A.—(1921) *Beitr. path. Anat.*, **78**, 161.
Teoh, T. B.—(1957) *J. Path. Bact.*, **73**, 451.
Trotter, W.—(1911) *Br. med. J.*, **2**, 1057.